# Math Anxiety and Financial Anxiety Predicting Individuals' Financial Management Behavior

**DOI:** 10.1155/2023/3131631

**Published:** 2023-06-02

**Authors:** Ziqiang Xin, Huiwen Xiao, Gege Lin

**Affiliations:** ^1^Department of Psychology, Renmin University of China, Beijing 100872, China; ^2^School of Sociology and Psychology, Central University of Finance and Economics, Beijing 100081, China

## Abstract

**Background:**

In managing finances, people need to process various financial texts containing math (e.g., amount of money and mathematical concepts) and financial information (e.g., funds and bonds). Such information could trigger anxiety related to math and finance; however, previous literature has rarely investigated the prediction role of contextual anxiety on financial management behavior. Therefore, the current study examined how math anxiety and financial anxiety are related to individuals' financial management behavior assessed by self-report and objective observation, respectively.

**Methods:**

Study 1 investigated 186 employees with the math anxiety scale, financial anxiety scale, and financial management behavior scale to explore how math anxiety and financial anxiety predicted financial management behavior. Study 2 used a “choice/no choice” paradigm to observe how the high (*n* = 50) and low (*n* = 53) financial anxiety groups chose (or avoided) between a math task and a finance task (as a measurement of financial avoidance).

**Results:**

Study 1 showed that financial anxiety fully mediated the negative relationship between math anxiety and financial management behavior and the mediating effect size was −0.24, 95%CI = [−0.34, −0.16]. And compared to math anxiety (*r* = −0.24, *p* < 0.01), financial anxiety (*r* = −0.45, *p* < 0.01) was a stronger negative predictor of financial management behavior. Study 2 revealed that, compared to people with low financial anxiety, those with high financial anxiety were 2.75 times more likely to choose financial avoidance.

**Conclusions:**

People's financial management behavior can be predicted by financial anxiety and math anxiety (especially the former), and the two types of anxiety seem to derive more from an irrational self-perception rather than actual ability. So, reducing financial anxiety and math anxiety should come first to motivate people to manage finances.

## 1. Introduction

Since the reform and opening up in China, the per capita disposable income of Chinese residents has grown at an average annual rate of 8.5%, and people have increasing money at their disposal [1]. At the same time, a large number of financial products have flooded the market, providing people with a wide variety of financial alternatives. Even so, many people are often reluctant to engage in financial activities such as stock and fund investments, credit spending, and debt management. This phenomenon has been explained by many economists and psychologists, who contend that people's subjective and objective financial knowledge, their perception regarding the complexity of financial decisions, and their unique personality traits are significant factors for individuals' reluctance to manage their finances [2–5]. However, researchers have neglected the influence of contextual anxiety on financial management behavior in financial activities. Actually, when individuals participate in financial activities, they are usually exposed to various financial texts containing financial and math information [6, 7], and they are required to process the financial texts and then make financial decisions. During the process, some people may experience “math anxiety” when they are first exposed to math information, and some people may experience “financial anxiety” when they read the financial information. These contextual anxieties in financial activities may interrupt individuals' current financial decisions. According to the above, the current study is aimed at exploring the predictive effects of math anxiety and financial anxiety on financial management behavior.

### 1.1. Concepts of Math Anxiety, Financial Anxiety, and Financial Management Behavior and Their Direct Relationship

Financial management behavior refers to the utilization and management of an individual's limited assets to maximize their utility or achieve wealth accumulation. It involves a series of steps, starting with being exposed to financial texts and ending with decision-making [8, 9]. In this process, individuals usually contact diverse financial texts involving math information, such as interest rates, compound interest, probabilities, and amounts of money. It has been evidenced that anxiety towards math information could drive people to avoid financial activities when faced with decisions requiring mathematical effort [10, 11]. Thus, math anxiety, which is defined as an anxious state that occurs when individuals confront figures or are required to solve math problems [12, 13], may be an important factor in individuals' financial management behavior. Previous literature about math anxiety typically focused on the educational domain. For instance, many studies revealed that people who have high math anxiety struggled with math tasks, devoted less time and energy to math learning, and even had the propensity to avoid math-related courses [14–16]. In contrast, the influence of math anxiety is not restricted to the context of math learning and teaching but can extend to a wide range of life situations, including counting changes in a store, reconciling restaurant bills, and calculating purchase prices [17, 18]. For example, Suri et al. [19] found that math anxiety not only increased tendency to make computational errors but also impaired cognitive ability to make numerical judgments. In a word, financial management behavior, as a direct application scenario of mathematics in real life, is likely to be influenced by math anxiety when processing and interpreting mathematical content [20].

In addition to numerical and quantitative information, financial texts that people are exposed to, of course, contain financial content, such as information about funds, bonds, and bills. Consequently, those who are in financial situations may experience financial anxiety, which ultimately affects their financial decision-making. The term “financial anxiety” in the present study refers to the fact that people frequently feel anxious and frightened when confronted with financial texts that have specific professional properties in situations where they need to make financial decisions. However, prior research typically defined financial anxiety as an uneasy attitude towards one's financial situation [21]. And prior extensive work concentrated on how anxiety about one's financial condition affected financial management behavior [22, 23]. For example, Grable et al. [23] found that those who were concerned about their poor financial condition did not seek out financial consultants and exhibited lesser financial management behavior. In addition, some previous studies also focus on the predictive effects of general anxiety on financial management behavior [24–26]. General anxiety is usually defined as an individual's disposition to worry about events, behaviors, and abilities [27]. It has been documented that those with high levels of general anxiety made more excessively conservative financial decisions, like avoiding investments [24, 26]. Unlike previous research, the current study focused primarily on contextual anxiety experienced by individuals in financial contexts [28]. This contextual anxiety towards financial activities might also serve as a more robust and direct predictor of financial management behavior.

### 1.2. Indirect Path of Math Anxiety Predicting Financial Management Behavior: The Mediating Role of Financial Anxiety

Even though both math anxiety and financial anxiety have the potential to discourage financial management behavior, they may act in different sequences. Theoretically, financial anxiety may directly decrease financial management behavior, whereas math anxiety may indirectly decrease financial management behavior through financial anxiety. This is attributed to the fact that, in terms of individual development, math anxiety typically arises during childhood, whereas financial anxiety usually emerges later in adolescence and adulthood [11, 12, 29]. For example, Yung et al. [30] found that college students' anxiety when taking finance courses was driven by courses' extensive use of complex mathematical materials. However, math anxiety and financial anxiety may also act simultaneously. Starting from the information processing process, we found that when people are involved in financial activities, they first go through an information input process where they are accessible with various forms of information, incorporating financial and mathematical content [7]. It is difficult to determine the sequence in which math anxiety and financial anxiety occur at this point. Therefore, the current study constructed a simultaneous prediction model and a mediation model to examine how math anxiety and financial anxiety predicted financial management behavior.

Although math anxiety and financial anxiety may affect financial management behavior in different sequences, the underlying psychological processes should be quite similar. This issue can be interpreted by the attentional control theory [31], which postulated efficient cognitive processing depending on the balance between the two attentional systems of a top-down goal-driven system and a bottom-up stimulus-driven system. However, anxiety can disrupt people's attentional systems. Concretely, when faced with an anxious situation, the stimulus-driven system dominates and the goal-driven system is functionally limited, resulting in more difficulty for them to resist the irrelevant stimuli and focus on task-relevant stimuli [32, 33]. As such, those people with high math anxiety and high financial anxiety may have more trouble focusing their attention on understanding and digesting financial and math information contained in financial texts, ultimately leading to a tendency to avoid financial activities.

### 1.3. Theoretical Framework and Purpose of the Present Study

**Figure 1:**
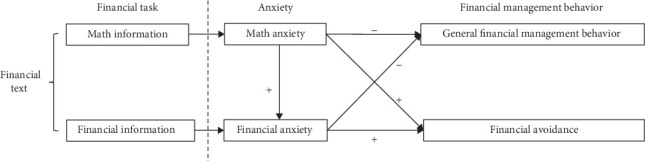
A hypothetical framework for “financial task, anxiety, and financial management behavior.”

Taken together, we propose a hypothetical framework of “financial task, anxiety, and financial management behavior” (Figure 1). In this framework, the main concepts are related as follows: (1) financial tasks usually contain both math (e.g., amount of money, mathematical concepts, and mathematical symbols) and financial information (e.g., financial concepts and financial terms), which could induce math anxiety and financial anxiety, respectively. (2) The predictive effects of the two anxieties on financial management behavior may have different sequences. One is the direct path, in which both anxiety directly decreases general financial management behavior or increases financial avoidance. The other is the indirect path, in which math anxiety indirectly decreases general financial management behavior or increases financial avoidance through financial anxiety. In other words, math anxiety is a distal factor whereas financial anxiety is a proximal factor.

As shown in Figure 1, the conceptual or a priori relationship between the variables is on the left side, while the relationship among the variables on the right side is needed to be verified. Thus, we aimed to examine the predictive effects of math anxiety and financial anxiety on personal financial management behavior through two studies. Study 1 used standardized scales to explore the relationship among math anxiety, financial anxiety, and financial management behavior. Since study 1 measured financial management behavior using a self-report approach, it might generate subjective bias. Study 2 used financial avoidance as an objective personal financial management behavior (a reverse measure) to further validate the effect of financial anxiety on financial management behavior at the behavioral level. The findings of the present study would contribute to a better understanding of how different types of contextual anxiety predict financial management behavior and offer suggestions for people to improve their financial management behavior.

## 2. Study 1: The Relationship among Math Anxiety, Financial Anxiety, and Financial Management Behavior

### 2.1. Method

#### 2.1.1. Participants

Because there were no relevant previous studies, we used a medium effect size (*f*^2^ = 0.15) to determine the sample size in study 1. G⁣^∗^Power 3.1 software analysis recommended that 107 participants were needed to achieve 95% power using linear multiple regression analysis. Therefore, a convenience sampling method was used to recruit 200 working employees, who came from state-owned enterprises, private enterprises, schools, hospitals, and other institutions. All participants filled in the informed consent form and then completed the scales. When the responses were gathered, a preliminary check was made, and then, those participants having many missing or duplicate options for the scale items were excluded. A total of 186 people (61% female) with ages ranging from 21 to 63 (*M*_age_ = 28.23, SD = 6.55) were obtained.  Table 1 summarizes the demographic features of the sample.

#### 2.1.2. Measurements

*(1) Math Anxiety Scale*. Math anxiety scale was adapted from Hunt et al. [34]. It consisted of 23 items with three subscales, and the situations for measurement were all math-related scenarios. The math evaluation anxiety subscale measures the degree of anxiety that individuals felt when being assessed in mathematics, such as taking a math quiz and answering mathematical questions in public (e.g., “Taking a math exam”). The social math anxiety subscale measures the level of anxiety that people experienced in mathematical activities in daily life (e.g., “Being asked to add up the number of people in each session”). The math observation anxiety subscale measures anxiety when presented with mathematical content (e.g., “Reading a math textbook”). Participants rated the degree of their anxiety on a 5-point Likert scale ranging from 1 (“not at all”) to 5 (“very much”), with higher scores indicating higher levels of math anxiety. Confirmatory factor analysis was conducted on the scale, and the model fit indicators met the acceptable criteria (*χ*^2^/*df* = 2.06, RMSEA = 0.08, CFI = 0.90, TLI = 0.89, and SRMR = 0.06), indicating that it had good construct validity. Cronbach's alpha coefficients for the total scale and its three subscales were 0.96, 0.94, 0.92, and 0.89, respectively.

*(2) Financial Anxiety Scale*. Financial anxiety scale (FAS) measures individuals' tendency to feel anxious when engaging in financial activities. The development of the scale was referred to the math anxiety scale, but the situations for measurement were based on financial activities (e.g., “Dealing with financial issues makes me so nervous that I have headache”). The initial scale was developed with 15 positive items, and it was rated on a 5-point Likert scale (1 = “does not describe me at all” and 5 = “describes me completely”), with higher scores indicating higher levels of financial anxiety. An exploratory factor analysis using principal component analysis and the maximum variance method was conducted on the initial financial anxiety scale. It was found that the factor loadings and commonalities for all questions were greater than 0.40, so all items were retained. There were two factors with eigenvalues greater than 1, and their total explained variance was 71.27%. One factor was named “emotional anxiety,” comprising a range of negative emotions related to financial management, such as worry, nervousness, helplessness, and unease. Another factor was “avoidance tendency” towards financial management, like being afraid of managing one's own finances or entrusting someone else to manage them. Cronbach's alpha coefficients for the total scale and its two subscales were 0.96, 0.96, and 0.88, respectively.

*(3) Financial Management Behavior Scale*. The Chinese version of the financial management behavior scale was translated from Dew and Xiao's [9], with minor adjustments to the items based on the actual situation in China. The scale consisted of 15 items with four dimensions, namely, savings and investment, insurance, cash management, and credit management. All of them represented “general financial management behavior” (e.g., “invested bonds, stocks, or mutual funds”). There were 12 items with positive scoring and 3 items with negative scoring. All items were rated on a 5-point Likert scale (1 = “never” and 5 = “always”), with higher scores representing more general financial management behavior. Confirmatory factor analysis was conducted on the scale, and the model fit indicators met the acceptable criteria (*χ*^2^/*df* = 2.33, RMSEA = 0.09, CFI = 0.92, TLI = 0.90, and SRMR = 0.07), indicating that it had good construct validity. Cronbach's alpha coefficients for the total scale and its four dimensions were 0.86, 0.84, 0.87, 0.72, and 0.72, respectively.

#### 2.1.3. Data Analysis

Using SPSS 25.0, first, the common method variance test was performed to examine the common method variance effect of the self-reported measures [35]. Second, the demographic differences in math anxiety, financial anxiety, and financial management behavior were examined. Third, we carried out descriptive statistics and Pearson's correlation analysis for all research variables. Fourth, Mplus 8.3 was employed to implement path analysis using maximum likelihood estimation to test two competing models. Due to the potential impact of occupation, marriage, monthly income level, and household registration on the outcome variable, they were considered as control variables. In model 1, both math anxiety and financial anxiety simultaneously and directly predicted financial management behavior. In model 2, financial anxiety mediated the relationship between math anxiety and financial management behavior.

### 2.2. Results

#### 2.2.1. Common Method Variance Test

Harman's single-factor test was performed. The results showed that the exploratory factor analysis for the three scales exhibited 10 factors with eigenvalues greater than 1, and the variance explained by the first factor was 35.00%, which was lower than the suggested standard of 40%. Therefore, there were no serious common method biases in the present study.

#### 2.2.2. Demographic Differences of Variables

To understand the demographic differences (gender, marriage, type of occupation, monthly income level, and household registration) in math anxiety, financial anxiety, and financial management behavior, independent *t*-tests and one-way ANOVAs were conducted. The results showed no significant gender differences in math anxiety, financial anxiety, and financial management behavior (*p* > 0.05). Those who worked in financial industries had significantly more financial management behavior than those who did not work in financial industries (*t*(184) = 3.25, *p* = 0.001, Cohen's *d* = 0.63), but there were no significant differences in math anxiety and financial anxiety between the two types of occupation (*p* > 0.05). Married people had more financial management behavior than unmarried people (*t*(184) = −3.38, *p* = 0.001, Cohen's *d* = 0.53), but there were no significant differences in math anxiety and financial anxiety between the married and unmarried (*p* > 0.05). Simultaneously, those with higher monthly income levels had less financial anxiety (*F*(2, 183) = 3.22, *p* = 0.04, *ƞ*^2^ = 0.03) and more financial management behavior (*F*(2, 183) = 6.38, *p* = 0.002, *ƞ*^2^ = 0.07), but there were no significant income level differences in math anxiety (*p* > 0.05). Those with urban household registration had significantly less financial anxiety (*t*(184) = 3.23, *p* = 0.001, Cohen's *d* = 0.47) and more financial management behavior than those with rural household registration (*t*(184) = 3.50, *p* = 0.001, Cohen's *d* = 0.53), while there was no significant difference in math anxiety between urban and rural household registration (*p* > 0.05).

#### 2.2.3. Descriptive Statistics and Correlations of Variables

Descriptive statistics of variables are shown in Table 2. Math anxiety was significantly positively correlated with financial anxiety (*r* = 0.58, *p* < 0.01); however, it was significantly negatively correlated with financial management behavior (*r* = −0.24, *p* < 0.01). Financial anxiety was also negatively associated with financial management behavior (*r* = −0.45, *p* < 0.01).

#### 2.2.4. Path Analysis

Path analysis was conducted to compare the two competing models. In model 1, both math anxiety and financial anxiety served as direct predictors of financial management behavior. The results showed that math anxiety was nonsignificantly associated with financial management behavior (*β* = 0.03, *p* = 0.71), whereas financial anxiety was significantly and negatively associated with financial management behavior (*β* = −0.42, *p* < 0.001). The results imply that the predictive effect of math anxiety on financial management behavior was overshadowed by financial anxiety, which suggests the presence of a possible mediation model.

**Figure 2:**
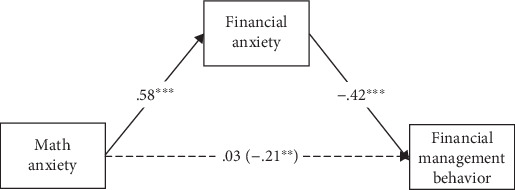
A mediation role of financial anxiety in the relationship between math anxiety and financial management behavior after excluding the impacts of control variables (⁣^∗∗^*p* < 0.01 and ⁣^∗∗∗^*p* < 0.001).

In model 2, the results showed that math anxiety significantly and positively predicted financial anxiety (*β* = 0.58, *p* < 0.001) and financial anxiety further significantly and negatively predicted financial management behavior (*β* = −0.42, *p* < 0.001), while the direct effect of math anxiety on financial management behavior was not significant (*β* = 0.03, *p* = 0.71). Although the indirect and direct effects have opposite symbols, the masking effect was excluded. Because the total effect of math anxiety on financial management behavior was significant (*β* = −0.21, *p* < 0.01), and the direct effect of math anxiety on financial management behavior was rendered insignificant with the inclusion of financial anxiety [36, 37]. In this model, financial anxiety played a fully mediating role in the relationship between math anxiety and financial management behavior, and the mediating effect size was −0.24. The results of bootstrap analysis showed that the indirect effect did not contain 0 between the upper and lower limits of the 95% bootstrap confidence interval (95%CI = [−0.34, −0.16]), indicating a statistically significant mediating effect of financial anxiety (Figure 2).

### 2.3. Discussion

This study was the first time to explore the predictive effect of math anxiety and financial anxiety on individuals' financial management behavior in a financial context. And it was found that financial anxiety fully mediated the relationship between math anxiety and financial management behavior, indicating that individuals' math anxiety induced by math information in financial texts increased their financial anxiety in financial activities, which in turn decreased financial management behavior. More importantly, compared to math anxiety, financial anxiety had a stronger negative predictive effect on financial management behavior. The findings imply that math anxiety and financial anxiety are, respectively, distal and proximal factors that influence financial management behavior. Given that, study 2 would concentrate on financial anxiety. Moreover, to improve the construct validity, financial avoidance was measured through an adaptation of the “choice/no choice” paradigm as an objective financial management behavioral indicator in study 2.

## 3. Study 2: Differences in Financial Avoidance between High and Low Financial Anxious People

### 3.1. Method

#### 3.1.1. Participants

Individuals with the top 27% and bottom 27% of financial anxiety scores in study 1 were recruited. A total of 103 people (60% female) with ages ranging from 21 to 63 (*M*_age_ = 28.07, SD = 7.10) were obtained. We used G⁣^∗^Power 3.1 software to ensure a sufficient sample size for study 2. Since there were no previous studies, we used a medium effect size (odds ratio = 2.5), and it recommended that 82 participants were needed to achieve 95% power using binary logistic regression. It suggested that the sample size of study 2 is sufficient.

#### 3.1.2. Design and Procedure

The independent variable was the financial anxiety level grouping (0 = low financial anxiety group and 1 = high financial anxiety group), and the dependent variable was financial avoidance (0 = no avoidance and 1 = avoidance). Participants were divided into a high financial anxiety group (*n* = 50, *M* = 3.51, SD = 0.43) and a low financial anxiety group (*n* = 53, *M* = 1.19, SD = 0.17) according to the financial anxiety scores. After grouping, participants were asked to choose twice between the math task and the Chinese language task, as well as the math task and the finance task through an adapted “choice/no choice” paradigm [38]. Their second choice was used as an indicator of individuals' financial avoidance. Finally, participants were required to respond to the task chosen for the second time (only the math task or finance task).

Both of the math task and the finance task contained ten problems that were self-designed. Each pair of math and finance problems is different only in cover stories rather than in mathematical essence, because both of them are the same in numbers, quantitative relationships, arithmetic operations, and answers. For example, in the math task, problem 1 is “Three tractors can cultivate 900 hectares of land in 3 days, so five tractors can cultivate _____ hectares of land in 6 days.” In the finance task, problem 1 is “Three vending machines can make a profit of 900 yuan in 3 days, so five vending machines can make a profit of _____yuan in 6 days.” Since people's ability to solve math or finance tasks might influence the relationship between financial anxiety and financial management behavior, it was necessary to exclude it. Therefore, we used an individual's correct answer score (one point for a correct answer) on either the math task or the finance task to represent his or her math problem-solving ability or financial problem-solving ability, with a theoretical range of scores from 0 to 10.

**Figure 3:**
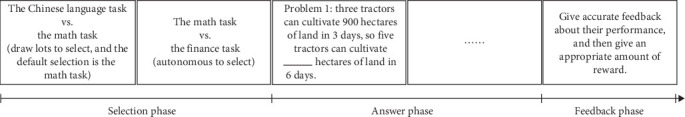
The research flowchart of study 2.

The entire study was designed in the form of a questionnaire. There were three stages: selection, answer, and feedback (see Figure 3). First, the participants were informed that it was a test of math ability or Chinese language ability, and then, they were asked to choose one of the two types of tasks to answer. If the participants fully answered the problems under the chosen task correctly, he/she would be rewarded with ¥10, while ¥1 would be deducted from ¥10 for each wrong question. Second, after informed rules, the participants were instructed to make the first choice between the math task and the Chinese language task through a random drawing process. It should be noted that all participants were initially assigned the math task by default. Subsequently, participants were provided with an opportunity to make a second choice between the math task and the finance task in an autonomous manner. In the second choice, if the participant chose the math task rather than the finance task, it was considered as financial avoidance. In the answer phase, all participants were given sufficient time to answer the questions under their chosen task (10 math problems or 10 finance problems). Finally, they were given accurate feedback about their problem-solving, and then, they received an appropriate amount of reward based on their performance.

#### 3.1.3. Data Analysis

Using SPSS 25.0, first, a 2^∗^2 chi-square test of independence was conducted to examine the characteristics of high and low financial anxiety groups on financial avoidance. Second, logistic regression was conducted with high and low financial anxiety groups as an independent variable, financial avoidance as a dependent variable, and gender, type of occupation, marriage, monthly income level, and household registration as control variables. Third, we separately used an independent *t*-test and Mann-Whitney *U* nonparametric test to examine the differences in the correct scores of math or finance tasks in the high and low financial anxiety groups, to exclude the effect of math problem-solving ability or financial problem-solving ability on financial avoidance.

### 3.2. Results

The results of a 2^∗^2 chi-square test of independence showed that there was a significant difference in financial avoidance between the high and low financial anxiety groups (*χ*^2^ = 4.61, *df* = 1, *p* = 0.03, *Ф* = 0.02). Specifically, the percentage of participants choosing financial avoidance in the high financial anxiety group (90%) significantly exceeded that in the low financial anxiety group (74%).

The result of logistic regression is shown in Table 3. Firstly, all control variables had a nonsignificant effect on financial avoidance. Second, there was a significant difference in financial avoidance between the high financial anxiety group and the low financial anxiety group (*B* = 1.32, Wald *χ*^2^ = 4.78, *p* = 0.03). Specifically, when compared to people with low financial anxiety, those with high financial anxiety were 2.75 times more likely to choose financial avoidance.

The differences in the correct scores of math or finance tasks in the different financial anxiety groups were examined. The results showed that for participants who chose the math task, there was no significant difference in the correct scores on the math task between the high financial anxiety group (*n* = 39, *M* = 2.33, SD = 1.95) and the low financial anxiety group (*n* = 45, *M* = 2.18, SD = 2.13) (*t*(82) = 0.35, *p* = 0.73, Cohen's *d* = 0.07). To be noted, participants in both the high and low anxiety groups scored relatively low on the math task. It may be due to the solution involving many computational processes, but the scoring procedure merely considered the final result, not the process performance. For participants who chose the finance task, being smaller in number (*n* = 19), we used the Mann-Whitney *U* nonparametric test. It revealed that participants in the high financial anxiety group (*n* = 5, *M* = 2.60, SD = 2.30) seemed to score lower on the finance task compared to participants in the low financial anxiety group (*n* = 14, *M* = 4.36, SD = 1.91). But this difference was not significant (*Z* = −1.45, *p* = 0.15). The above results suggested that the differences in financial avoidance for the high and low financial anxiety groups were not explained by math problem-solving ability or financial problem-solving ability. Nevertheless, the exclusion of the impact of people's abilities needs to be further validated in the future.

### 3.3. Discussion

Study 2 used the choice (whether to avoid) between a math task and a finance task as a reverse measurement for financial management behavior, to examine the predictive effects of financial anxiety on financial avoidance. The results indicated that those with high financial anxiety tend to choose financial avoidance more than those with low financial anxiety, and this result was not affected by the people's math problem-solving ability or financial problem-solving ability. It reconfirmed the results of study 1. However, compared to study 1, study 2 did not find any differences in gender, type of occupation, marriage, monthly income level, and household registration. It may be explained by the fact that financial avoidance represents only one form of financial management behavior and the smaller sample size of study 2. In summary, study 2 increased the construct validity of the study by utilizing objective behavior instead of employing the self-report method.

## 4. General Discussion

The present study targeted the working population and explored the relationship among math anxiety, financial anxiety, and financial management behavior in financial situations through self-reported and behavioral level measures. Study 1 found that financial anxiety fully mediated the relationship between math anxiety and financial management behavior, and financial anxiety was a greater predictor of financial management behavior compared to math anxiety. Study 2 found that, compared to people with low financial anxiety, those with high financial anxiety were more likely to choose financial avoidance.

### 4.1. The Framework of Math Anxiety and Financial Anxiety Predicting Financial Management Behavior

**Figure 4:**
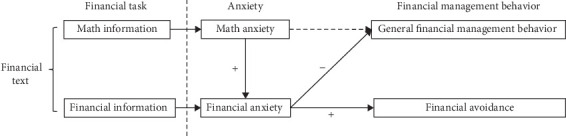
A confirmed framework for “financial task, anxiety, and financial management behavior.”

Although both direct and indirect paths between math anxiety and financial management behavior are assumed in the anticipated framework in Figure 1, study 1 only confirmed the indirect path (Figure 4). It means that math anxiety can be generalized to financial activities, but it is a distant influencing factor for financial management behavior compared to financial anxiety. The results are in line with some related studies, which suggest that math anxiety emerged earlier than financial anxiety, and it tended to induce individuals to feel financial anxious to learn financial courses [12, 29, 30]. Therefore, when people encounter math information in financial texts, they typically produce higher math anxiety. Then, this anxiety can further reinforce their financial anxiety when confronted with financial information, ultimately preventing people from engaging in financial activities [39, 40].

To conclude, according to the two studies, financial anxiety was a more direct and powerful suppressor of financial management behavior than math anxiety (study 1), and it made people avoid financial activities (study 2), as illustrated in Figure 4. Thoughtfully, study 2 showed that the high and low financial anxiety groups did not appear to differ fundamentally in their math problem-solving ability or financial problem-solving ability, suggesting that this anxiety triggered by financial texts was derived more from an irrational self-perception rather than actual ability. This finding is in contrast to the previous research, which claimed that insufficient financial knowledge and skills can predict people's anxiety in financial situations [25, 41].

The adverse effects of both math anxiety and financial anxiety on financial management behavior were in different sequences, but they should be common in their psychological mechanisms. According to related anxiety theories, anxiety usually induces people to quickly capture and focus on threatening stimuli or to interpret ambiguous stimuli as threatening [42–44], and then, anxious people frequently have a bias toward the overestimation of the probability of negative events or threat, further resulting in a tendency to take a safer action, such as retreat, avoidance, and passive behavior [45–47]. For example, Smith et al. [48] found that individuals with high anxiety were more inclined to perceive ambiguous surroundings as unstable, which decreased their propensity to take risks. Similarly, a survey by Kim et al. [49] also found that relative to healthy participants, pathologically anxious participants frequently exhibited an overestimation of risk in their daily lives. More than that, highly anxious people typically underestimate their ability to cope with such threats [50], leading to their avoidance of events. Therefore, according to the above, the reason for individual financial avoidance is probably that when people experience math anxiety or financial anxiety, they may view financial texts or financial problems as threat stimuli and overestimate their difficulty, thus taking financial avoidance. Future research could further test this assumption.

### 4.2. Theoretical and Clinical Implications

The current study made several important theoretical and clinical contributions. First, the present findings have confirmed a model to explain how contextual anxiety that people are confronted with in financial activities affects financial management behavior. And these results evidence that financial anxiety mediates the relationship between math anxiety and financial management behavior. Second, the results have direct practical implications. In the current study, we found that people's contextual anxiety in financial activities seems the result of irrational self-perceptions rather than a genuine inadequacy of abilities. Therefore, guiding people to adjust their self-perceptions and reducing unnecessary mathematical calculations and financial terminologies in financial texts will help them to reduce anxiety and promote their enjoyment of financial activities.

### 4.3. Limitations and Future Directions

There are some limitations in the current study. First, the study did not manipulate math anxiety or financial anxiety to examine their causal relationship with outcome variables. The current results are still a correlation or prediction relationship. Second, the measurement of financial avoidance in study 2 was assessed by the individual's choice between math and finance tasks. Although it provided an objective behavioral indicator, it was still different from real financial management behavior. These deficiencies can serve as breakthroughs for future research. Third, previous studies have shown that general anxiety can affect financial management behavior [24, 26], but we did not control it in the present study.

## 5. Conclusions

Both financial anxiety and math anxiety (especially the former) could predict people's financial management behavior, and financial anxiety fully mediated the negative relationship between math anxiety and financial management behavior. Moreover, people with high financial anxiety were more likely to choose financial avoidance than those with low financial anxiety. The two types of anxiety seem to derive more from an irrational self-perception rather than actual ability. So, reducing financial anxiety and math anxiety should come first to motivate people to manage finances.

## Figures and Tables

**Table 1:** Demographic differences in math anxiety, financial anxiety, and financial management behavior.

Demographic variables	Math anxiety	Financial anxiety	Financial management behavior
Gender			
Male (*n* = 73)	1.87 ± 0.83	2.23 ± 0.97	3.55 ± 0.77
Female (*n* = 113)	1.93 ± 0.80	2.19 ± 0.93	3.36 ± 0.69
Occupation type			
Financial industry employees (*n* = 33)	1.67 ± 0.66	2.00 ± 0.99	3.80 ± 0.69
Nonfinancial industry employees (*n* = 153)	1.96 ± 0.83	2.25 ± 0.93	3.36 ± 0.71
Marriage			
Unmarried (*n* = 123)	1.88 ± 0.79	2.22 ± 0.94	3.31 ± 0.72
Married (*n* = 63)	1.97 ± 0.85	2.19 ± 0.95	3.68 ± 0.67
Monthly income level			
¥0~4999 (*n* = 52)	1.95 ± 0.84	2.30 ± 0.94	3.21 ± 0.62
¥5000~9999 (*n* = 86)	1.96 ± 0.84	2.32 ± 0.99	3.42 ± 0.71
¥10,000 and above (*n* = 48)	1.77 ± 0.72	1.91 ± 0.81	3.71 ± 0.79
Household registration			
Urban (*n* = 118)	1.86 ± 0.74	2.04 ± 0.84	3.57 ± 0.75
Rural (*n* = 68)	1.99 ± 0.91	2.49 ± 1.05	3.20 ± 0.63

**Table 2:** Descriptive statistics and correlations of variables (*N* = 186).

Variables	*M*	SD	1	2	3
Math anxiety	1.91	0.81	—		
Financial anxiety	2.21	0.94	0.58⁣^∗∗^	—	
Financial management behavior	3.44	0.73	-0.24⁣^∗∗^	-0.45⁣^∗∗^	—

⁣^∗∗^*p* < 0.01.

**Table 3:** Comparison of financial avoidance for those in the high and low financial anxiety groups (*N* = 103).

Independent variables	*B*	SE	Wald	Exp(*B*)
Gender				
Male				1.00
Female	0.29	0.55	0.27	1.33
Occupation type				
Financial industry employees				1.00
Nonfinancial industry employees	-0.58	0.77	0.57	0.56
Marriage				
Unmarried				1.00
Married	-0.17	0.71	0.06	0.84
Household registration				
Rural				1.00
Urban	0.27	0.58	0.21	1.31
Monthly income level				
Under ¥2000				1.00
¥2000~¥4999	0.29	0.64	0.20	1.34
¥5000~¥9999	0.31	0.79	0.15	1.36
Financial anxiety group				
Low financial anxiety group				1.00
High financial anxiety group	1.32	0.60	4.78⁣^∗^	3.75

⁣^∗^*p* < 0.05.

## Data Availability

The data and materials that support the findings of this research are available from the corresponding author upon request.

## References

[1] National Bureau of Statistics of China Residents’ living standards continue to rise, and the quality of consumption has improved significantly - the fourth in a series of reports on the achievements of economic and social development in the 40 years of reform and opening up. http://www.stats.gov.cn/ztjc/ztfx/ggkf40n/201808/t20180831_1620079.html.

[2] Rai K., Dua S., Yadav M. (2019). Association of financial attitude, financial behaviour and financial knowledge towards financial literacy: a structural equation modeling approach. *FIIB Business Review*.

[3] Wang J., Yu Y. (2017). The impact of financial literacy on undergraduates’ participation in stock market: the mediating role of confidence level. *Psychology: Techniques and Applications*.

[4] Xiao H., Xin Z. (2022). Financial literacy is better than income to predict happiness. *Journal of Neuroscience, Psychology, and Economics*.

[5] Xiao J. J., Tang C., Shim S. (2009). Acting for happiness: financial behavior and life satisfaction of college students. *Social Indicators Research*.

[6] Xin Z., Yang Z. (2023). Provincial marketization and individual financial literacy in China. *Journal of Neuroscience, Psychology, and Economics*.

[7] Zhang H., Xin Z., Wu X. (2020). The development of financial capacity test for Chinese citizens. *Psychology: Techniques and Applications*.

[8] Deacon R. E., Firebaugh F. M. (1988). *Family Resource Management: Principles and Applications*.

[9] Dew J., Xiao J. J. (2011). The financial management behavior scale: development and validation. *Journal of Financial Counseling and Planning*.

[10] Choe K. W., Braxton J., Rozek C. S., Berman M. G., Beilock S. L. (2019). Calculated avoidance: math anxiety predicts math avoidance in effort-based decision-making. *Science Advances*.

[11] Krinzinger H., Kaufmann L., Willmes K. (2009). Math anxiety and math ability in early primary school years. *Journal of Psychoeducational Assessment*.

[12] Maloney E. A., Beilock S. L. (2012). Math anxiety: who has it, why it develops, and how to guard against it. *Trends in Cognitive Sciences*.

[13] Richardson F. C., Suinn R. M. (1972). The mathematics anxiety rating scale: psychometric data. *Journal of Counseling Psychology*.

[14] Ashcraft M. H., Krause J. A. (2007). Working memory, math performance, and math anxiety. *Psychonomic Bulletin Review*.

[15] Foley A. E., Herts J. B., Borgonovi F., Guerriero S., Levine S. C., Beilock S. L. (2017). The math anxiety-performance link. *Current Directions in Psychological Science*.

[16] Paechter M., Macher D., Martskvishvili K., Wimmer S., Papousek I. (2017). Mathematics anxiety and statistics anxiety. Shared but also unshared components and antagonistic contributions to performance in statistics. *Frontiers in Psychology*.

[17] Silk K. J., Parrott R. L. (2014). Math anxiety and exposure to statistics in messages about genetically modified foods: effects of numeracy, math self-efficacy, and form of presentation. *Journal of Health Communication*.

[18] Skagerlund K., Lind T., Stromback C., Tinghog G., Vastfjall D. (2018). Financial literacy and the role of numeracy-how individuals' attitude and affinity with numbers influence financial literacy. *Journal of Behavioral and Experimental Economics*.

[19] Suri R., Monroe K. B., Koc U. (2013). Math anxiety and its effects on consumers’ preference for price promotion formats. *Journal of the Academy of Marketing Science*.

[20] Choi S. S., Taber J. M., Thompson C. A., Sidney P. G. (2020). Math anxiety, but not induced stress, is associated with objective numeracy. *Journal of Experimental Psychology: Applied*.

[21] Shapiro G. K., Burchell B. J. (2012). Measuring financial anxiety. *Journal of Neuroscience, Psychology, and Economics*.

[22] Gignac G. E., Gerrans P., Andersen C. B. (2023). Financial literacy mediates the effect between verbal intelligence and financial anxiety. *Personality and Individual Differences*.

[23] Grable J. E., Heo W., Rabbani A. (2014). Financial anxiety, physiological arousal, and planning intention. *Journal of Financial Therapy*.

[24] Gambetti E., Giusberti F. (2019). Personality, decision-making styles and investments. *Journal of Behavioral and Experimental Economics*.

[25] Grable J. E., Archuleta K. L., Ford M. R., Kruger M., Gale J., Goetz J. (2020). The moderating effect of generalized anxiety and financial knowledge on financial management behavior. *Contemporary Family Therapy*.

[26] Shih T. Y., Ke S. C. (2014). Determinates of financial behavior: insights into consumer money attitudes and financial literacy. *Service Business*.

[27] Xie F., Xin Z., Chen X., Zhang L. (2019). Gender difference of Chinese high school students’ math anxiety: the effects of self-esteem, test anxiety and general anxiety. *Sex Roles*.

[28] Yu Y. J., Rha J. Y., Yeo J. S., Ko E., Kim S. Y. (2019). Consumers’ anxiety about financial transactions. *Financial Planning Review*.

[29] Sorvo R., Koponen T., Viholainen H. (2017). Math anxiety and its relationship with basic arithmetic skills among primary school children. *British Journal of Education Psychology*.

[30] Yung A., Chau S., Ho W. (2015). Measuring anxiety and self-efficacy in studying finance-course for students in business-related disciplines in Hong Kong. *International Journal of Arts & Sciences*.

[31] Eysenck M. W., Derakshan N., Santos R., Calvo M. G. (2007). Anxiety and cognitive performance: attentional control theory. *Emotion*.

[32] Ashcraft M. H., Kirk E. P. (2001). The relationships among working memory, math anxiety, and performance. *Journal of Experimental Psychology: General*.

[33] Hopko D. R., Ashcraft M. H., Gute J., Ruggiero K. J., Lewis C. (1998). Mathematics anxiety and working memory: support for the existence of a deficient inhibition mechanism. *Journal of Anxiety Disorders*.

[34] Hunt T., Clark-Carter D., Sheffield D. (2011). The development and part validation of a U.K. scale for mathematics anxiety. *Journal of Psychoeducational Assessment*.

[35] Aulakh P. S., Gencturk E. F. (2000). International principal-agent relationships: control, governance and performance. *Industrial Marketing Management*.

[36] Baron R. M., Kenny D. A. (1986). The moderator-mediator variable distinction in social psychological research: conceptual, strategic, and statistical considerations. *Journal of Personality and Social Psychology*.

[37] Judd C. M., Kenny D. A. (1981). Process analysis: Estimating mediation in treatment evaluations. *Evaluation Review*.

[38] Siegler R. S., Lemaire P. (1997). Older and younger adults’ strategy choices in multiplication: testing predictions of ASCM using the choice/no-choice method. *Journal of Experimental Psychology: General*.

[39] Morsanyi K., Busdraghi C., Primi C. (2014). Mathematical anxiety is linked to reduced cognitive reflection: a potential road from discomfort in the mathematics classroom to susceptibility to biases. *Behavioral and Brain Functions*.

[40] Park J. J., Sela A. (2018). Not my type: why affective decision-makers are reluctant to make financial decisions. *Journal of Consumer Research*.

[41] Kadoya Y., Khan M. S. R. (2018). Can financial literacy reduce anxiety about life in old age?. *Journal of Risk Research*.

[42] Eysenck M. W., Calvo M. G. (1992). Anxiety and performance: the processing efficiency theory. *Cognition and Emotion*.

[43] Gasper K., Clore G. L. (1998). The persistent use of negative affect by anxious individuals to estimate risk. *Journal of Personality and Social Psychology*.

[44] Olatunji B. O., Ciesielski B. G., Armstrong T., Zhao M., Zald D. H. (2011). Making something out of nothing: neutral content modulates attention in generalized anxiety disorder. *Depression and Anxiety*.

[45] Beesdo-Baum K., Jenjahn E., Höfler M., Lueken U., Becker E. S., Hoyer J. (2012). Avoidance, safety behavior, and reassurance seeking in generalized anxiety disorder. *Depression and Anxiety*.

[46] Bishop S. J., Gagne C. (2018). Anxiety, depression, and decision making: a computational perspective. *Annual Review of Neuroscience*.

[47] Salmeto A. L., Hymel K. A., Carpenter E. C., Brilot B. O., Bateson M., Sufka K. J. (2011). Cognitive bias in the chick anxiety-depression model. *Brain Research*.

[48] Smith A. R., Ebert E. E., Broman-Fulks J. J. (2016). The relationship between anxiety and risk taking is moderated by ambiguity. *Personality and Individual Differences*.

[49] Kim M., Kim S., Lee K.-U., Jeong B. (2020). Pessimistically biased perception in panic disorder during risk learning. *Depression and Anxiety*.

[50] Ladouceur R., Blais F., Freeston M. H., Dugas M. J. (1998). Problem solving and problem orientation in generalized anxiety disorder. *Journal of Anxiety Disorder*.

